# Complications Associated with Uterine Artery Embolisation for Fibroids

**DOI:** 10.1155/2012/290542

**Published:** 2011-11-24

**Authors:** Maria Memtsa, Hayden Homer

**Affiliations:** ^1^Department of Obstetrics & Gynaecology, Institute for Women's Health, University College London and University College London Hospitals NHS Foundation Trust, London NW1 2BU, UK; ^2^Mammalian Oocyte & Embryo Research Laboratory, Division of Biosciences, Department of Cell and Developmental Biology, University College London, London WC1E 6BT, UK; ^3^Reproductive Medicine Unit, EGA Wing, Institute for Women's Health, University College London and University College London Hospitals NHS Foundation Trust, London NW1 2BU, UK

## Abstract

Uterine artery embolisation (UAE) is a relative newcomer to the mainstream treatment modalities available for fibroid-related problems. The efficacy of UAE is indisputable and has been shown to be comparable to hysterectomy in the short term in large-scale trials. Moreover, compared with hysterectomy, UAE is less invasive, carries a superior risk profile, and, importantly, preserves the uterus. UAE therefore offers patients symptom relief whilst at the same time retaining reproductive potential. Notably however, although women can have successful pregnancies following UAE, it is becoming increasingly evident that pregnancies after UAE are more risky especially during the early stages. Long-term outcome data from randomised trials involving UAE have very recently become available and show that whilst high satisfaction rates previously identified during early-stage followup are sustained, one notable drawback is a substantial risk of reintervention. It remains to be seen how this facet of UAE will impact on its future uptake.

## 1. Introduction

The occlusion of the vascular supply of uterine fibroids, uterine artery embolisation (UAE), was first described in 1995 [1] and is now firmly established as a minimally invasive organ-preserving approach for treating symptomatic uterine fibroids. Short term followup data from randomised controlled trials have established UAE as a safe and effective alternative to hysterectomy for alleviating fibroid-related disorders such as menorrhagia and pressure symptoms [2]. However, a fuller appreciation of the longer term efficacy and complications of UAE is only just becoming apparent. Therefore, although gaining in popularity, it is important that patients be apprised of these emerging data so that they will be well equipped to make informed decisions regarding the suitability of this treatment modality. This paper will summarise the reported side effects, complications, and symptom recurrence rate, not only in the short term but, as 5-year followup data are starting to emerge, also in the long term. We will also review the available evidence regarding the adverse effects of UAE on fertility and pregnancy outcome.

## 2. Technical Aspects and Periprocedural Side Effects of UAE

UAE exploits the differential recovery ability between fibroids and normal uterine tissue in response to ischaemia that is induced at the arteriolar level [3]. Using a femoral artery approach, the uterine artery is catheterised thereby allowing for the injection of embolic particles, most often made of poly-vinyl alcohol (PVA), that disrupt the blood supply to the fibroid (hence the alternative term *Uterine Fibroid Embolisation* or UFE). As fibroids derive their blood supply from both sides in nearly all cases, the procedure is repeated bilaterally altogether taking up to around 90 minutes to complete [4].

Pelvic pain is the most commonly reported side effect of UAE. The most likely causes are ischaemia of the fibroid and temporary ischaemia of the myometrium. The onset of the pain can be immediate, and it usually lasts about 12 hours. Hence, an overnight stay with adequate pain relief is usually required after the procedure. More severe pain usually subsides within the first 24 hours but moderate pain may persist for up to 2-3 days. Nausea typically accompanies the pain and usually requires pharmacological management [4].

Bruno and colleagues reported on the recovery of 99 patients who underwent UAE [5]. All patients in their study had an overnight stay and received a combination of intravenous NSAID (ketorolac) and patient-controlled administration of intravenous morphine. Based on a visual analogue scale of 0–10 (where 0 is no pain and 10 worst imaginable pain), the mean pain scores over the first 24 hours and first week were 3.03 and 4.83, respectively. Recovery was fairly fast, with the vast majority (94%) of patients missing less than 10 days of work [5].

Another frequent morbidity of UAE is postembolisation syndrome, which includes fever, pain, malaise, and nausea and lasts from a few hours to a few days. The syndrome typically occurs after embolisation of any solid organ and is thought to be an immune-mediated response. It has been reported to occur in ~50% of patients but is easily controlled with analgesics, antipyretics, and anti-inflammatories [6].

## 3. Adverse Events Associated with UAE

Large multicentre trials have consistently reported low rates of adverse events associated with UAE. Adverse effects can be categorised into major, minor, and severe, with the majority of them presenting within the first month after procedure [6].

The largest series to have assessed adverse effects, the FIBROID registry, enrolled 3 000 patients [7] and reported a 0.66% rate of in-hospital major adverse events and a 4.8% rate within the first 30 days following discharge.  Table 1 summarises the important complications in the acute (≤24 hours after UAE), subacute (>24 hours to up to 1 week after UAE) and chronic (>1 week postoperatively) phases. Although pain is often a nonspecific side effect, it is imperative for the treating clinician to be aware that pain that is more intractable could be a harbinger of a more sinister underlying complication such as endometritis, pelvic abscess formation, or endometrial ischaemic necrosis that would require clinical and laboratory evaluation and possibly further imaging [6].

A frequent complication of UAE reported in up to 3% of cases is the vaginal expulsion of an infarcted fibroid often requiring further intervention [8]. This complication is thought to arise when UAE is used in the presence of either submucosal fibroids or intramural fibroids that have a substantial submucosal component. Imaging in the form of a contrast-enhanced MRI is helpful in evaluating the size of the infarcted fragment and its degree of extrusion into the uterine cavity as well as the extent of tissue that remains adherent to the surrounding myometrium. In cases in which a partially infarcted fibroid remains firmly attached to the myometrium, hysteroscopic resection or dilatation and curettage may be required.

Uterine infection is a more serious complication and patients may present with severe pain of sudden onset, vaginal discharge, and/or bleeding. Infection needs to be aggressively treated as it may lead to systemic sepsis and, occasionally, could require hysterectomy [9]. Very rarely, death due to overwhelming sepsis has also been described [10]. Fortunately, it is a relatively rare complication having been reported in less than 1% of cases [9].

Another rare but also potentially fatal complication of UAE is pulmonary embolism, which is thought to be attributed to a transient hypercoagulable state similar to, but not as severe as, that seen with surgical procedures [4, 11]. The incidence has been estimated to be 1 in 400 [12] making it prudent to undertake a thromboprophylaxis risk assessment prior to UAE.

Off-target organ embolisation has also been documented and is most likely to affect the ovaries (discussed in more detail later). Although the vaginal artery usually arises as a separate branch of the internal iliac artery, it may at times share a common trunk with the uterine artery or form anastomoses within the broad ligament. Under such circumstances, the vagina becomes vulnerable to embolisation-induced ischaemia [6] resulting in sexual dysfunction and/or dyspareunia [13].

Despite all of the above, the evidence from multicentre trials consistently shows that the rate of major adverse events following UAE is significantly lower than those related to more conventional surgical interventions such as myomectomy and hysterectomy. In the REST trial, a UK-based multicentre trial that recruited 157 women with symptomatic fibroids, the major complication rate for UAE in the first year, was 12% compared to 20% for surgical patients [14]. Similarly, in the HOPEFUL trial, a UK trial involving 18 NHS trusts, the complication rate was 4.5% for UAE compared with 14.8% for surgically managed patients [15]. In the EMMY trial, a randomised controlled trial conducted in The Netherlands involving 177 patients, when blood transfusion was included in the calculations, the major complication rate in the surgically treated group was 14.5% while the major complication rate assigned to the UAE group was only 1.3% [16].

## 4. Fertility after UAE

There is accumulating evidence that women can and do conceive after UAE. However, it is becoming increasingly apparent that UAE is associated with a greater risk of pregnancy complications. On the basis of this, a desire to maintain fertility is listed as a relative contraindication for UAE in the joint Standards of Practice recently set out by the Cardiovascular and Interventional Radiological Society of Europe and the Society of Interventional Radiology [17].

The only randomised controlled trial to have addressed fertility after UAE compared pregnancies following myomectomy (laparoscopic and open) with those following UAE in women who were desirous of future pregnancies [18]. Results from this trial favoured myomectomy in the short term (up to 2 years after treatment) suggesting a benefit of laparoscopic myomectomy over UAE in terms of likelihood of conceiving (77.5% versus 50%) and a relative risk of not getting pregnant following UAE of 2.22 [18].

Fertility would also be severely jeopardised if off-target effects of UAE were to compromise ovarian function. As discussed previously, off-target effects on the ovarian vasculature arising from uteroovarian collaterals are well documented. This results in ovarian ischaemia, depletion of ovarian follicles, and a risk of premature menopause. This catastrophic effect is mostly seen amongst patients over 45 years of age [19] perhaps because their pretreatment ovarian reserve is likely to have been low. Notably however, younger women are not immune to this complication [20]. In a study measuring FSH levels before and after procedure, 6% of women experienced transient postmenopausal symptoms including amenorrhoea, hot flushes, and elevated FSH levels, most of which resolved by 10 months after UAE [21].

## 5. Risk of Miscarriage after UAE

Although mainly observational in nature, one of the most striking aspects regarding the impact of UAE on pregnancy outcome pertains to miscarriage rates. Amongst women conceiving following UAE, spontaneous miscarriage rates range from 18.2% [22] to as high as 64.3% [23] (Table 2). Strikingly, based on currently available data, the cumulative risk of miscarriage after UAE is ~35% (Table 2), threefold higher than rates in the general population [24, 25].

Two parameters known to independently influence miscarriage rates are maternal age and fibroid location. Thus, older women are exquisitely susceptible to miscarriage due to their increased risk of having pregnancies with abnormal chromosomal compliments [26, 27] whereas fibroid subtypes which distort the uterine cavity (submucosal fibroids) have the most deleterious effect on miscarriage. A recent review that used pooled data from previously published series compared the risk of miscarriage after UAE with that in women who had untreated fibroids and matched groups for age and fibroid location [24]. Even after controlling for these two parameters, the risk of miscarriage after UAE was ~35% compared to ~16% in fibroid-containing pregnancies (odds ratio 2.8, 95% confidence interval 2.0–3.8) [24]. These data regarding UAE are counterintuitive to the notion that treatment of fibroids should reduce miscarriage rates and point to a detrimental impact of the UAE procedure on the uterine milieu.

It is unclear why UAE imposes additional risks for miscarriage over and above those known to be associated with fibroids [24]. One possibility is that endometrial ischaemia following UAE permanently alters the quality of potential implantation sites, making it hostile to the early conceptus. Fibroid migration and distortion of the endometrial contour has also been implicated as a causative factor for miscarriage. In line with this, amongst 51 women who underwent UAE for intramural fibroids and were followed up by hysteroscopy, intrauterine protrusion of the fibroid remnant was documented in 37% of cases, whilst adhesions were reported in 14% and in 10% of instances there was a communication between the myoma and the endometrial cavity [28]. Indeed, only 37% of women had a hysteroscopically normal cavity [28]. The above factors, along with other currently unknown downstream effects of UAE, likely contribute to the increased risk of early pregnancy failure.

## 6. Obstetric Outcomes after UAE 

Although the foregoing data clearly identify early pregnancy as a vulnerable stage following UAE, later stages of pregnancy are not immune. The parameters used to evaluate the impact of UAE on obstetric performance have included preterm birth rates, mode of delivery, fetal presentation, fetal growth restriction, and postpartum haemorrhage. Recent studies have amalgamated these parameters from observational and cohort studies, together comprising over 200 pregnancies after UAE [24, 25] (Table 3). Compared with women having fibroid uteri who had received no intervention, it is evident that the rates of Caesarean section delivery and postpartum haemorrhage (PPH) are significantly increased after UAE [24, 25].

As acknowledged previously, important caveats need to be borne in mind when interpreting these comparative data involving UAE. Notably, the control group includes women who, although possessing fibroids, were asymptomatic making it likely that they, on average, had smaller and fewer fibroids. In addition, other confounding factors that could potentially impact on pregnancy outcome such as body mass index (BMI) and parity were inconsistently reported and could not therefore be controlled for. Also, the increased risk of Caesarean delivery following UAE could reflect patient preference or the clinician's reluctance in advising a trial of labour in women considered to be at high risk rather than a robust indication for abdominal delivery due to effects directly ascribable to UAE *per se*. Similarly, the increased risk of PPH may be a consequence of the increased rates of Caesarean delivery rather than any predisposition to uterine haemorrhage brought about by embolisation. Reassuringly, there was no increase in risks of preterm delivery, malpresentation, or fetal growth restriction in the pregnancies that successfully negotiated the early stages of gestation [24, 25].

## 7. Risk of Recurrence after UAE

A very important aspect of UAE that has recently become apparent with the publication of longer-term followup data is a substantial risk of requiring reintervention. Although not a complication *per se*, this is a significant revelation that patients need to be cognisant of as it could sway their decision-making towards more radical treatment modalities such as hysterectomy if the desire to avoid reintervention is a high priority.

Long-term data from observational studies confirm that although UAE is effective, there is a recurrence rate of about 20% at 5 years [4], defined as subsequent need for hysterectomy, myomectomy, or repeat embolisation. Recently, the 5-year results from the EMMY and REST randomised controlled trials were published and point to an even higher rate of reintervention. In the EMMY trial of UAE (88 patients) versus hysterectomy (89 patients) for symptomatic uterine fibroids, 23 of 81 (28.4%) of UAE patients subsequently underwent a hysterectomy due to insufficient improvement of symptoms [29]. More recently, long-term followup results were reported for the REST trial, during which women were randomised to either UAE (106 patients) or surgery (42 hysterectomies and 9 myomectomies), and show a very similar 5-year intervention rate of 32% in the UAE group versus only 4% in the surgery group [30]. One very notable consequence of this degree of reintervention was that the initial cost benefit of UAE over surgery that was observed at 12 months was substantially reduced at 5 years, making both treatments cost neutral [30].

## 8. Conclusion

UAE for fibroids is a safe and effective minimally invasive treatment that promises substantial improvement in symptom control for women seeking a uterine-sparing therapy. However, it remains to be seen whether the longer-term need for reintervention in almost one-third of cases influences patient uptake of the procedure. Moreover, with an increasingly financially conscious health service, the longer-term dissipation of the short-term cost benefits of UAE over surgery could influence how widely this therapeutic option is offered. For women desirous of preserving future fertility, careful counselling of the potential risks associated with ensuing pregnancies is paramount. The available evidence, albeit very limited, suggests that myomectomy may be the treatment of choice in such women providing surgery is a viable option. Further prospective research on the impact of UAE on reproductive performance is needed before more definitive conclusions can be drawn.

## Figures and Tables

**Table 1:** 

Acute Phase	Subacute and chronic phase
Pain	Pain
Postembolisation syndrome	Transient or permanent amenorrhoea
Sepsis	Endometritis
Bleeding	Urinary retention
Groin haematoma	Passage of fibroid tissue
Contrast reaction	Delayed contrast reaction
Vasovagal response	Tuboovarian abscess
Pulmonary embolus	Uterine necrosis and rupture

**Table 2:** 

Study	*N*	Miscarriage (%)
Goldberg and Pereira, 2006 [31]	51	12 (23.5)
Pron et al., 2005 [22]	22	4 (18.2)
Walker and McDowell, 2006 [32]	53	17 (32.1)
Holub et al., 2007 [23]	24	14 (58.3)
Mara et al., 2008 [18]	14	9 (64.3)
Hirst et al., 2008 [33]	34	15 (44.1)

Cumulative data	198	71/198 (35.9)

**Table 3:** 

Obstetric complication	General population (%)	After UAE (%)
Postpartum haemorrhage	6	13.8
Preterm delivery	10	17.3
Caesarean delivery	22	67.5
IUGR	10	8
Malpresentation	5	9.4
